# Stage-resolved gene drivers of pancreatic ductal adenocarcinoma progression and therapeutic vulnerabilities

**DOI:** 10.1016/j.isci.2026.115164

**Published:** 2026-02-27

**Authors:** Eduardo Chuluyan, Brice Chanez, Analia Meilerman, Carla Remolins, Andrea Paes de Lima, Kevin Matamoros, Claudio Incardona, Diego Guerrieri, Gustavo Kohan, Florencia Gottardo, Martin Ledesma, Juan Garona, Carlos Davio, Agustin Yaneff, Ana Sahores, Daniel Grasso, Maria Noe Garcia, Daniela Papademetrio, Maria Eugenia Pasqualini, Luis Sarotto, Felix Gabriel Lezcano, Jonathan Garnier, Analia Pasqua, Oscar Mazza, Pascal Hammel, Nicolas Fraunhoffer, Nelson Dusetti, Juan Iovanna

**Affiliations:** 1 Programa Franco-argentino de Estudio del Cáncer de Páncreas, Buenos Aires, Argentina; 2 Centro de Estudios Farmacológicos y Botánicos (CEFYBO), Facultad de Medicina, Universidad de Buenos Aires-CONICET, Buenos Aires, Argentina; 3 Departamento de Microbiología, Parasitología e Inmunología, Facultad de Medicina, Universidad de Buenos Aires, Buenos Aires, Argentina; 4 Joint Lab INSERM-CONICET-UBA, Buenos Aires, Argentina; 5 Institut Paoli-Calmettes, Marseille, France; 6 Aix-Marseille University, INSERM U1068, CNRS UMR 7258, Institut Paoli-Calmettes, Centre de Recherche en Cancérologie de Marseille (CRCM), Parc Scientifique et Technologique de Luminy, Marseille, France; 7 Departamento de Patología, Hospital de Clínicas “José de San Martín”, Universidad de Buenos Aires, Buenos Aires, Argentina; 8 Fundación Gador, Buenos Aires, Argentina; 9 Bernardino Rivadavia Hospital, Buenos Aires, Argentina; 10 Unidad de Investigaciones Biomédicas en Cáncer (IBioCAN), Centro de Medicina Traslacional (CEMET), Hospital El Cruce (HEC), Buenos Aires, Argentina; 11 Centro de Oncología Molecular y Traslacional (COMTra), Universidad Nacional de Quilmes (UNQ), Buenos Aires, Argentina; 12 Instituto de Investigaciones Farmacológicas (ININFA), Facultad de Farmacia y Bioquímica, Universidad de Buenos Aires – CONICET, Buenos Aires, Argentina; 13 Instituto de Estudios de la Inmunidad Humoral (IDEHU), CONICET, Universidad de Buenos Aires, Buenos Aires, Argentina; 14 Departamento de Ciencias Biológicas, Facultad de Farmacia y Bioquímica, Universidad de Buenos Aires, Buenos Aires, Argentina; 15 Cátedra de Inmunología, Departamento de Microbiología, Inmunologia, Biotecnología y Genética, Facultad de Farmacia y Bioquímica, Universidad de Buenos Aires, Buenos Aires, Argentina; 16 Centro de Investigaciones en Biomedicina Traslacional (CIBiMeT), Hospital de Alta Complejidad del Bicentenario Esteban Echeverría - CONICET, Buenos Aires, Argentina; 17 Instituto de Investigaciones en Ciencias de la Salud, Consejo Nacional de Investigaciones Científicas y Técnicas and Facultad de Ciencias Médicas-Universidad Nacional de Córdoba, Ciudad Universitaria, Córdoba 5000, Argentina; 18 Departamento de Cirugia, Hospital de Clínicas “José de San Martín”, Universidad de Buenos Aires, Buenos Aires, Argentina; 19 Gastroenterology Department, Hospital Italiano de Buenos Aires, Buenos Aires, Argentina; 20 General Surgery Department, Hospital Italiano de Buenos Aires, Buenos Aires, Argentina; 21 Paul Brousse Hospital, Paris, France

**Keywords:** immunology, bioinformatics, computational bioinformatics, systems biology, cancer systems biology, cancer, omics, transcriptomics

## Abstract

Pancreatic ductal adenocarcinoma shows early dissemination, stromal remodeling, and therapy resistance, but how tumor programs co-evolve with immune and stromal changes across stages remains unclear. We built a stage-resolved transcriptomic atlas using bulk RNA sequencing of FFPE samples from 443 untreated tumors spanning resectable, locally advanced, primary metastatic, and liver metastatic disease. Integrative modeling identified ten transcriptional programs with monotonic dynamics during progression. Early stages were enriched for epithelial differentiation and immune-stimulatory signals. Advanced stages showed increased cytoskeletal remodeling, vesicular trafficking, oxidative stress responses, and mitochondrial metabolism. Pathway analysis revealed enhanced PI3K-AKT-mTOR signaling and MYC target engagement in metastatic disease. Immune deconvolution showed loss of CD8^+^ T cells, dendritic cells, and M1 macrophages. Compact gene signatures stratified stage and survival and generalized to TCGA and ICGC cohorts. Functional assays confirmed greater invasiveness, altered redox balance, and mitochondrial dependence in advanced disease, suggesting stage-associated metabolic vulnerabilities.

## Highlights

• Stage-resolved map of 443 PDACs reveals ten dynamic programs across progression
• KRAS rewiring to PI3K-AKT-mTOR with MYC drives late-stage adaptation
• Shift to OXPHOS/redox and cytoskeletal remodeling (e.g., COQ7 and EPAS1)
• Assays show increased invasiveness and mitochondrial dependence in PDAC

## Graphical abstract

**Figure:**
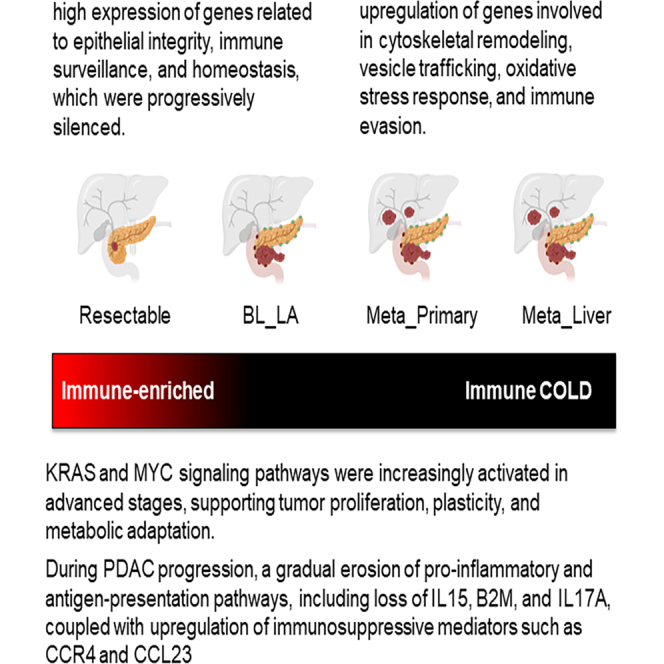

## Introduction

Cancer development can be broadly divided into two major phases: transformation and progression.^1^^,^^2^^,^^3^^,^^4^ The transformation phase involves the initial molecular events that convert normal cells into malignant ones. This process is primarily driven by mutations in oncogenes, tumor suppressor genes, and DNA repair genes, often accompanied by epigenetic alterations, such as DNA methylation and histone modifications. These changes disrupt normal cellular regulation, promoting uncontrolled proliferation, and survival. Transformation is frequently initiated by environmental exposures (e.g., smoking, radiation, and carcinogens) or inherited genetic predispositions. By overriding cell-cycle checkpoints and apoptotic pathways, transformed cells gain the capacity for autonomous growth. This early phase lays the groundwork for tumorigenesis by dismantling intrinsic control mechanisms. Understanding the transformation phase is critical for identifying early oncogenic drivers and developing preventive or early intervention strategies aimed at halting cancer before it progresses.^5^

The second phase, cancer progression, involves the evolution of tumors toward increasing malignancy and aggressiveness.^6^^,^^7^^,^^8^ During this phase, cancer cells acquire additional mutations and adapt to the tumor microenvironment, becoming more invasive, and resistant to therapy.^6^ Key hallmarks of progression include angiogenesis (the formation of new blood vessels to support tumor growth),^9^ immune evasion (the ability to escape immune detection),^10^ and most critically, metastasis (the spread of cancer cells to distant organs).^11^^,^^12^ Metastatic tumors are often life-threatening and are characterized by increased invasiveness, apoptosis resistance, and the ability to colonize distant, often hostile, tissues.^13^^,^^14^ Understanding tumor progression is essential for tackling advanced cancer. This stage defines how tumors grow, invade, and eventually spread. Dissecting these mechanisms provides crucial insight into therapeutic and metastatic dissemination, and informs the development of treatments aimed not only at the primary tumor but also at preventing metastasis and overcoming drug resistance.^15^

In this study, we sought to define stage-associated transcriptional programs underlying PDAC progression using a large cohort of clinically annotated, treatment-naive human tumors spanning resectable, borderline/locally advanced, and metastatic disease, including liver metastases. By integrating bulk RNA sequencing with unsupervised clustering, pathway activity inference, and computational immune and stromal deconvolution, we aimed to map coordinated changes in tumor-intrinsic signaling, metabolic adaptation, and microenvironmental remodeling across clinically defined stages. In addition, we explored whether selected transcriptional predictions could be functionally supported using patient-derived PDAC cultures. Although our analyses are primarily correlative, this stage-resolved framework provides a systems-level view of PDAC progression and enables identification of compact gene signatures associated with disease stage and outcome, generating testable hypotheses for future mechanistic and therapeutic studies.

## Results and discussion

### Cohort characteristics

**Figure 1:**
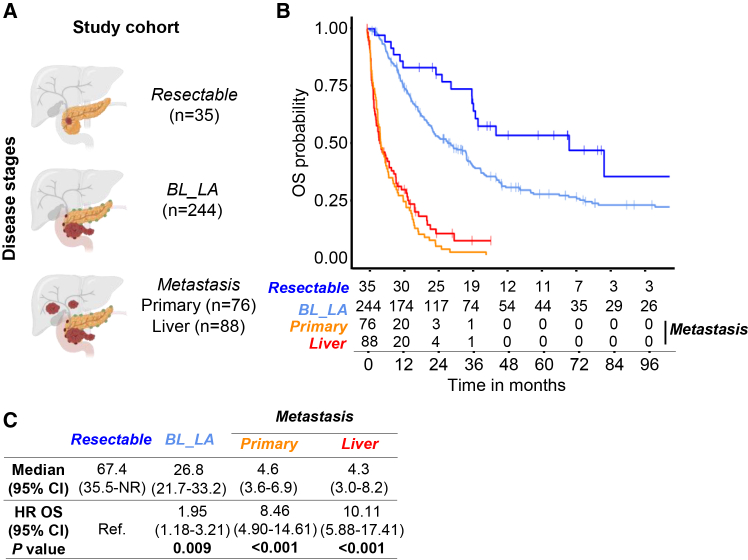
Overall survival stratified by tumor stage in patients with PDAC (A) A total of 443 patients with PDAC were included in the study and stratified as resected, borderline-locally advanced (BL_LA) and metastatic at the time of obtention of the PDAC sample. The distribution was as follows: Resected (*n* = 35), BL_LA (*n* = 244), and metastatic (*n* = 164). Among the metastatic cases, 76 samples were obtained from the primary pancreatic tumor, while 88 samples were derived from liver metastases. (B) Kaplan-Meier curves depicting overall survival (OS) of each group of patients. Median OS with 95% confidence intervals (CI) by group: operated 67.4 months (95% CI: 35.5-not reached); BL-LA, 26.8 months (95% CI: 21.7–33.2); Metastatic-primary tumor, 4.6 months (95% CI: 3.6–6.9); liver_meta, 4.3 months (95% CI: 3.0–8.2). (C) Hazard ratios (HR) for death derived from a Cox proportional hazards model using resectable as reference: BL_LA, HR = 1.95 (95% CI: 1.18–3.21; *p* = 0.009); Metastatic-primary tumor, HR = 8.46 (95% CI: 4.90–14.61; *p* < 0.001); liver_meta, HR = 10.11 (95% CI: 5.88–17.41; *p* < 0.001).

We analyzed 443 patients diagnosed with PDAC, stratified by clinical stage at the time of sample collection: resected (*n* = 35), borderline/locally advanced (BL_LA; *n* = 244), and metastatic (*n* = 164). Among metastatic cases, 76 samples were from primary pancreatic tumor and 88 from liver metastases (Figure 1A). Both metastatic subgroups were diagnosed at the same disease stage, making them clinically comparable. Kaplan-Meier survival analysis revealed significant stage-dependent differences in overall survival (OS). Median OS was 67.4 months (95% CI: 35.5-not reached) for resected patients, 26.8 months (95% CI: 21.7–33.2) for BL_LA, 4.6 months (95% CI: 3.6–6.9) for metastatic patients with primary tumor samples, and 4.3 months (95% CI: 3.0–8.2) for those with liver metastases (Figure 1B). Multivariable Cox regression confirmed increasing risk of death with advancing stage. Using resected patients as reference, the hazard ratio (HR) for BL_LA was 1.95 (95% CI: 1.18–3.21; *p* = 0.009), for metastatic-primary was 8.46 (95% CI: 4.90–14.61; *p* < 0.001), and for metastatic-liver was 10.11 (95% CI: 5.88–17.41; *p* < 0.001) (Figure 1C). These findings underscore the prognostic power of tumor stage in PDAC. The median overall survival (OS) observed for patients with resectable PDAC in our dataset (67.4 months) is longer than typically reported in large prospective cohorts (e.g., 24–36 months in CONKO-001 and PRODIGE-24). This discrepancy likely reflects several cohort-specific factors. First, the resectable cases included in this transcriptomic dataset were predominantly derived from high-volume tertiary centers with stringent surgical selection criteria and complete R0 resections, which are associated with improved long-term survival. Second, because the cohort integrates both institutional and public datasets, survival estimates may be influenced by censored cases and by the inclusion of patients alive at last follow-up, thereby slightly overestimating median OS. Finally, transcriptomic studies tend to preferentially include specimens of higher RNA integrity and adequate tumor content, which introduces a mild selection bias toward clinically favorable cases. These considerations are now explicitly discussed to clarify the characteristics and potential survival advantage of the resectable subset.

### Differential expression of cluster C1 reveals candidate genes for tumor progression and biomarker discovery

**Figure 2:**
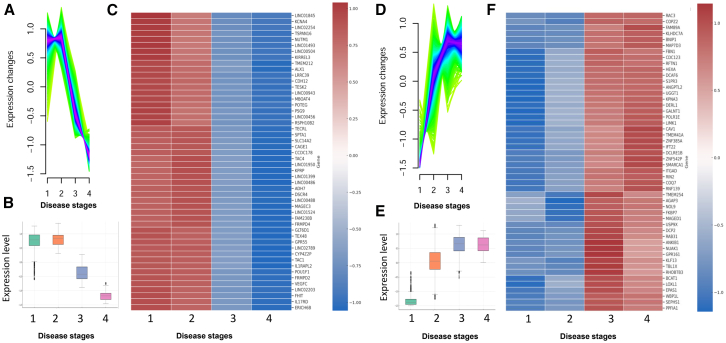
Transcriptomic trajectories during PDAC progression reveal two distinct gene expression clusters Unsupervised clustering of gene expression profiles across PDAC progression stages identified two major gene expression modules showing opposing dynamic patterns. Cluster 1 (left panel): This cluster comprises genes progressively downregulated from early to advanced disease stages. (A) The top ridge plot illustrates individual gene trajectories, showing consistent negative slopes across stages. (B) The boxplot summarizes the average expression trend of cluster 1 genes, highlighting a statistically significant reduction in expression as tumors progress toward metastasis. (C) The heatmap presents the top 50 representative genes from cluster 1, with normalized expression values (*Z* score scaled) across samples. Cluster 2 (right panel): This cluster includes genes that are progressively upregulated during tumor progression. (D) The ridge plot shows increasing gene expression from localized to metastatic stages. (E) The boxplot confirms a monotonic increase in average expression of this cluster across tumor stages, with peak expression in liver_meta. (F) The heatmap displays the top 50 genes from cluster 2. Gene expression data were normalized and scaled (row-wise *Z* score). Genes included in each cluster were selected based on their significant and consistent monotonic expression changes (adjusted *p* < 0.05) throughout PDAC progression. These results suggest that PDAC evolution is accompanied by coordinated activation and repression of gene programs, likely contributing to metastatic competence. 1 = resected; 2 = BL_LA; 3 = primary and 4 = liver_meta.

In this study, we compared RNA expression profiles from PDAC patients across disease stages: resected, BL_LA, and metastatic (including primary_meta and liver_meta samples) to identify genes associated with tumor progression. We focused on cluster C1, selected for its pronounced transcriptional shift across stages (Figure 2A), and prioritized the top 50 genes with the highest absolute fold change between early-stage (resected and BL_LA) and metastatic tumors. Quantitative expression analysis revealed a consistent, stage-dependent downregulation of C1 genes, with the sharpest decline occurring in metastatic disease. The complete list is shown in Table S1. Boxplot analysis confirmed this trend, showing a significant reduction in median expression levels across stages (resected: 0.73 ± 0.33; BL_LA: 0.80 ± 0.22; primary_meta: −0.36 ± 0.27; liver_meta: −1.18 ± 0.14) (Figure 2B). A heatmap visualization highlighted the coordinated suppression of cluster C1 genes during disease progression (Figure 2C), suggesting a loss of biological programs related to epithelial maintenance, immune regulation, and tumor suppressive signaling.

Hierarchical clustering further supported the existence of a coherent gene module, highly expressed in early-stage tumors and silenced in metastasis. This pattern aligns with a homeostatic transcriptional signature progressively lost during PDAC progression (Figure 2C). Several genes in cluster C1 are established regulators of epithelial structure and differentiation. For example, CDH12, a type II cadherin involved in cell-cell adhesion implicated in maintaining epithelial integrity, is downregulated during EMT and promotes invasiveness in multiple tumor types.^16^ Similarly, SPTA1, a cytoskeletal spectrin, and TESK1, an actin-regulating kinase, showed reduced expression in metastasis, suggesting roles in preserving cytoskeletal architecture and limiting motility.^17^^,^^18^ The tumor suppressor FHIT, also silenced in advanced stages, has been linked to genomic instability and apoptotic resistance in PDAC.^19^ In parallel, IL17RD, a negative regulator of MAPK signaling and inflammation, and SLC14A2, involved in epithelial osmotic homeostasis, were preferentially expressed in non-metastatic samples, reinforcing the link between cluster C1 and epithelial integrity and inflammatory restraint.^20^^,^^21^ Notably, this cluster also contained transcripts associated with neuroendocrine signaling (e.g., TAC1 and TAC4), whose loss may reflect impaired cellular communications and differentiation during PDAC evolution.^22^ Cancer-testis antigens MAGEC3^23^ and CAGE1,^24^ typically associated to late-stage disease, were unexpectedly enriched in early-stage tumors. Their coordinated silencing across progression stages may suggest roles beyond immune recognition, possibly linked to early tumor suppression or epigenetic regulation. A substantial portion of cluster C1 also contained several uncharacterized long non-coding RNAs (lncRNAs), including LINC00943, LINC00456, LINC00504, LINC01493, and LINC01845. These lncRNAs showed strong co-expression with the aforementioned protein-coding transcripts and exhibited clear stage-specific downregulation. Although functionally unannotated, their pattern suggests potential roles as *cis*-regulatory elements or scaffolds modulating chromatin accessibility and transcriptional networks.^25^^,^^26^ Their consistent suppression in metastasis supports their relevance as candidate tumor-suppressive lncRNAs, and novel biomarkers for PDAC progression.

### Identification of candidate driver genes associated with PDAC progression

To identify robust candidate genes representing the transcriptional reprogramming across PDAC stages, we applied a multi-step filtering and integration strategy. First, we performed differential expression analysis comparing resectable, locally advanced, and metastatic samples, retaining genes with |log_2_ FC| > 1 and FDR <0.01. Second, stage-dynamic genes were grouped by Mfuzz soft clustering, and those with high membership scores (>0.7) in clusters showing monotonic up- or downregulation across stages were selected. Third, we integrated these genes with pathway enrichment results (GSEA/GO) to prioritize those belonging to key biological processes (cytoskeletal remodeling, metabolic adaptation, and immune regulation). From this refined list, we built a LASSO-Cox regression model to identify genes most strongly associated with survival and stage progression, using 10-fold cross-validation to prevent overfitting. The top 15 genes retained by the final LASSO model were defined as candidate drivers. Their expression trends were validated in independent datasets (TCGA-PAAD and ICGC PACA-AU), and their protein-level relevance was confirmed using the human protein atlas (HPA) immunohistochemistry database. These genes represent key transcriptional nodes potentially mediating the transition from early to late PDAC stages.

### Upregulation of cluster C2 genes suggests roles in tumor progression and metastatic adaptation

**Figure 3:**
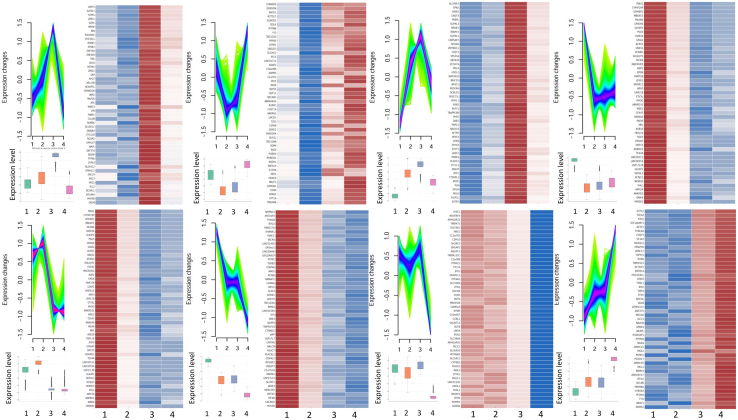
Transcriptomic modules clusters 3 to 10 reveal diverse expression dynamics during PDAC progression Unsupervised clustering of gene expression profiles across PDAC stages identified 10 gene expression clusters. Clusters 1 and 2 are shown in Figure 2; clusters 3 through 10 are shown here. Each cluster panel includes: A ridge plot (top left) illustrating the gene-wise expression trajectories across tumor stages. A boxplot (bottom) summarizing the average expression of all genes in the cluster per tumor stage. A heatmap (top right) displaying the top genes within each cluster, with expression values normalized (row-wise *Z* score). The clusters exhibit a range of transcriptional dynamics: clusters 3, 5, and 9 show non-monotonic patterns, such as transient activation or repression at intermediate stages, potentially reflecting stage-specific regulatory events. Clusters 4, 6, and 10 display progressive downregulation, indicating gene programs that are silenced during metastatic progression. Clusters 7 and 8 show gradual upregulation, suggestive of late-stage or metastasis-associated gene activation. These modules define the transcriptional architecture of PDAC progression and highlight stage-specific regulatory programs that may underlie tumor aggressiveness and metastatic competence. 1 = resected; 2 = BL_LA; 3 = primary and 4 = liver_meta.

We next investigated the expression dynamics of cluster C2 to identify genes dysregulated during PDAC progression. The 50 genes with the highest absolute fold change between early-stage and metastatic tumors were selected, representing transcripts most dynamically altered during disease evolution. Unlike cluster C1, which showed progressive silencing, cluster C2 exhibited a clear and consistent increase in expression from resected to metastatic stages (Figure 2D). This trend was confirmed by boxplot analysis (resected: −1.32 ± 0.19; BL_LA: 0.50 ± 0.45; primary_meta: 0.63 ± 0.34; liver_meta 0.63 ± 0.26) (Figure 3E) and by a heatmap visualization (Figure 2F), which highlighted coordinated transcriptional activation across advanced disease. These findings suggest that cluster C2 genes contribute to metastatic adaptation, supporting processes such as invasion, survival, and immune evasion. The complete list is shown in Table S2.

Several upregulated genes are directly involved in cytoskeletal remodeling and cell motility. Notably, RAC3, a Rho GTPase, regulates actin cytoskeleton dynamics and PI3K/AKT signaling. Its progressive upregulation across stages supports its role in promoting migration and invasion.^27^ MAP7D3, a microtubule-stabilizing protein, likely facilitates directional movement and intracellular transport in metastatic cells.^28^ Other transcripts are involved in vesicle trafficking and organelle homeostasis. COPZ2 (Golgi integrity), RAB31 (vesicle transport), and BNIP1 (ER-mitochondrial coordination) were elevated in metastatic tumors,^29^^,^^30^^,^^31^ suggesting an adaptation to heightened secretory and biosynthetic demands. Genes involved in hypoxia response and redox regulation were also enriched. EPAS1 (HIF-2α), a central regulator of angiogenesis and metabolic adaptation under low oxygen, was upregulated in advanced stages.^32^ SEPHS1 and COQ7, essential for selenoprotein and ubiquinone biosynthesis respectively, may contribute to oxidative stress tolerance.^33^^,^^34^ Stress survival pathways were also activated. USP9X, a deubiquitinase involved in proteostasis, supports cell survival under metabolic duress and is frequently altered in PDAC. NUAK1, an AMPK-related kinase, dirves KRAS-dependent tumor growth and may aid metabolic rewiring during metastasis.^35^^,^^36^ Cluster 2 also included genes associated with extracellular matrix (ECM) remodeling and angiogenesis. LOXL1, promotes collagen crosslinking and matrix stiffening, contributing to PDAC desmoplasia.^37^ ANGPTL2, a pro-angiogenic glycoprotein, also modulates immune signaling.^38^ FBN1, CAV1, and S1PR3 regulate stromal architecture, vascular signaling, and cell adhesion, reinforcing their roles in shaping a permissive microenvironment for invasion and metastasis.^39^^,^^40^ Finally, transcriptional and epigenetic regulators were prominently represented. SMARCA1 (chromatin remodeling), TBL1X (co-repressor complex component), and DCAF6 (co-regulator recruitment) suggest extensive gene regulatory reprogramming. The inclusion of KLF13, a transcription factor linked to EMT and immune modulation, underscores the plasticity and immune adaptation occurring in late-stage PDAC.^41^

Together, these findings position cluster C2 as a core gene module diving PDAC progression and metastatic competence, highlighting candidates for biomarker development and therapeutic targeting.

### Distinct expression dynamics of clusters C3 to C10 define divergent regulatory programs during PDAC progression

To elucidate transcriptional alterations associated with advanced PDAC, we analyzed the top 50 most dynamically regulated genes across clusters C3 to C10. Genes were ranked by their maximal expression range across the four progression stages. A significant subset of these genes, including COQ9, COX6A1, and NDUFA13, are involved in mitochondrial electron transport and redox balance. This indicates a metabolic shift toward oxidative phosphorylation (OXPHOS) during late-stage PDAC, consistent with reports that mitochondrial respiration promotes survival and metastatic colonization under nutrient-deprived microenvironments.^42^ Several genes, such as KDM5B, NCOR1, and TBL1X, encode epigenetic regulators linked to histone modification, chromatin remodeling, and nuclear receptor co-repression. These factors are known to repress differentiation-associated genes and enhance cell plasticity.^43^ For instance, KDM5B, a histone H3K4 demethylase, has been shown to silence epithelial identity and facilitate phenotypic switching in both pancreatic and breast cancers.^44^ Other genes, including DERL1, PSMA2, and USP9X, are key components of the ER-associated degradation (ERAD) and the ubiquitin-proteasome pathways. Their expression suggests increased proteotoxic stress adaptation in metastatic PDAC cells. These pathways support chemoresistance and immune evasion.^45^ USP9X, in particular, is essential for PDAC tumorigenesis in KRAS-driven models.^46^ Finally, transcripts such as RAC3, LIMK1, and NUAK1 point to activation of Rho-GTPase signaling and actin cytoskeleton regulation, key processes for migration and invasion. RAC3 enhances mesenchymal traits in PDAC, while NUAK1 supports cytoskeletal tension and anoikis resistance.^47^ This cytoskeletal remodeling is consistent with the emergence of a migratory, invasive phenotype in metastasis-prone tumors. To further characterize the metabolic reprogramming inferred from the transcriptional programs, we performed an in silico metabolic flux activity analysis based on the RECON2 human metabolic network. Using ssGSEA to estimate pathway-level activity scores, we observed a stage-dependent metabolic shift: early-stage clusters (C2-C4) were enriched in glycolytic and pentose phosphate pathways, whereas late-stage clusters (C6-C8) showed increased activity in oxidative phosphorylation, fatty acid β-oxidation, and amino acid degradation. These findings indicate that PDAC progression involves a transition from glycolytic dependence to mitochondrial oxidative metabolism, consistent with enhanced metabolic flexibility in metastatic tumors. The complete list is shown in Tables S3, S4, S5, S6, S7, S8, S9, and S10.

### Expression dynamics of KRAS and MYC target genes during PDAC progression

PDAC is defined by its near-universal dependence on oncogenic KRAS, which functions as the central signaling hub from tumor initiation through metastatic dissemination. Expression profiling across resected tumors, locally advanced lesions, and metastatic sites reveals dynamic regulation of canonical KRAS pathway genes, reflecting stage-specific reconfiguration of downstream signaling. Early in disease, proliferative drive is mediated predominantly through the RAF-MEK-ERK cascade, with effectors such as RAF1, BRAF, and MAPK1 transmitting strong mitogenic signals.^48^ As tumors progress toward metastatic competence, activation of the PI3K-AKT-mTOR axis, involving PIK3CA, PIK3CB, AKT3, and MTOR, becomes increasingly evident, supporting cell survival, biosynthetic growth, and adaptation to nutrient-limiting environments. The altered expression of regulatory components such as PTPN11, NF1, and RASA1 further illustrates how KRAS activity is sustained by weakening of negative feedback loops.

Running in parallel, MYC emerges as a transcriptional amplifier that converts KRAS-derived signaling inputs into broad transcriptional programs. Targets encoding replication licensing factors (MCM family, PCNA, and RRM1/2), cell-cycle regulators (CDK4/6 and CCND1/2), and mitotic drivers (AURKA, PLK1, and BIRC5) are progressively upregulated, providing the machinery necessary to sustain high proliferative turnover.^49^^,^^50^ In addition, MYC-driven transcriptional induction of ribosomal proteins, translation initiation factors, and elongation factors ensures the translational capacity required to support KRAS-driven growth. Metabolic enzymes such as ENO1, PKM, LDHA, and SLC2A1, also among MYC targets, are strongly induced during progression, complementing the metabolic reprogramming governed by the PI3K-AKT-mTOR branch of KRAS signaling.

**Figure 4:**
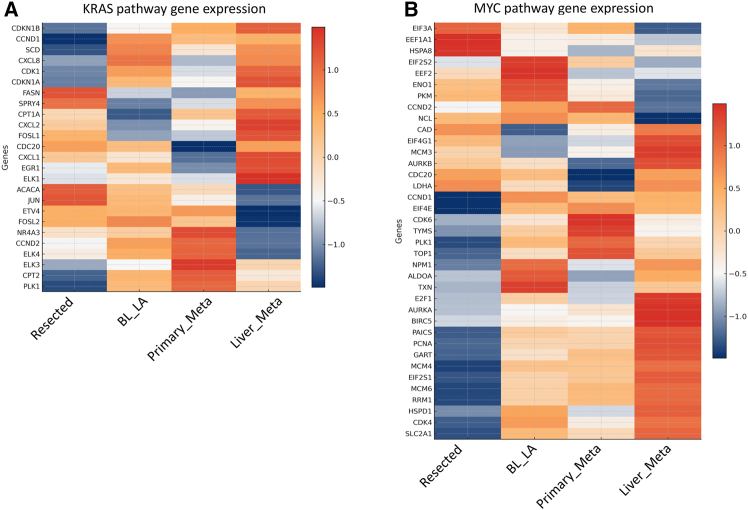
Dynamic regulation of KRAS and MYC target genes during PDAC progression Heatmaps display the relative expression (*Z* score normalized) of canonical downstream target genes of KRAS (A) and MYC (B) across four stages of PDAC progression. KRAS targets (left panel): genes involved in the MAPK and PI3K pathways (e.g., RAF1, PIK3CA, MAPK1, and BRAF) as well as transcriptional regulators (JUN and ETV4) display heterogeneous expression dynamics. Some genes (JUN, ETV4, and BRAF) peak at intermediate stages and are repressed in metastatic disease, while others (SPRY4 and EGFR) exhibit stage-specific modulation or oscillatory patterns. MYC targets (right panel): A distinct subset of MYC-regulated genes associated with proliferation (CCND1, CDK4, CDK6, and PCNA), metabolism (SLC2A1 and LDHA), and ribosome biogenesis (NCL and NPM1) shows progressive upregulation during tumor progression, with maximal expression frequently observed in advanced or metastatic stages. For example, PCNA, CDK4, and SLC2A1 exhibit consistent activation in metastasis. Color scale reflects standardized gene expression values (*Z* scores), with red indicating upregulation and blue downregulation relative to the cohort mean. These data highlight the differential regulation of KRAS and MYC effector programs throughout PDAC evolution, suggesting that MYC-driven transcriptional activation may play an increasingly dominant role in late-stage disease.

Together, these observations illustrate a cooperative oncogenic circuit in which KRAS provides the upstream instructive signals while MYC enforces the transcriptional reprogramming that enables their execution. KRAS orchestrates kinase cascades that rewire cellular signaling toward proliferation and metabolic adaptation, and MYC translates these inputs into a gene expression program that equips cells for continuous division, ribosome biogenesis, and glycolytic flux (Figure 4). KRAS pathway genes display an early surge in MAPK-driven effectors during the resected and locally advanced stages, establishing a proliferative baseline, followed by a progressive enrichment of PI3K-AKT-mTOR components in primary and liver metastases, consistent with the metabolic and survival demands of dissemination. In contrast, MYC targets show a more gradual and sustained increase across all stages, with replication and ribosome biogenesis genes elevated early, and metabolic and mitotic regulators further amplified in metastatic lesions. This temporal pattern highlights a division of labor: KRAS rapidly initiates proliferative and adaptive signaling, while MYC progressively consolidates these inputs into a durable transcriptional state that supports persistent growth and metastatic fitness.

### Immune recruitment genes: Suppression and redirection

**Figure 5:**
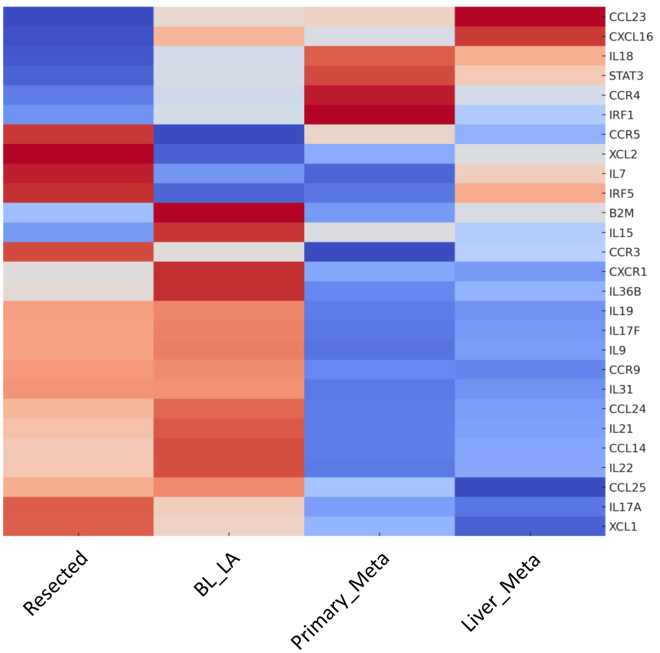
Differential expression profile of immune-regulatory genes within the tumor microenvironment The heatmap displays *Z* score normalized expression levels of selected cytokines, chemokines, transcription factors, and receptors (e.g., IL18, STAT3, CCR4, IRF5, and CXCL16) during PDAC progression. Hierarchical clustering based on similarity in expression patterns. The color scale ranges from blue (lower relative expression) to red (higher relative expression). This analysis highlights distinct inflammatory and immune-modulatory gene signatures that may play key roles in shaping the tumor microenvironment.

We generated a heatmap to visualize the expression patterns of immune-related genes, including interleukins, chemokines, their receptors, and immunomodulatory factors, throughout PDAC progression. The heatmap revealed distinct clusters separating genes that were progressively downregulated from those upregulated as disease advanced (Figure 5).

#### Progressive loss of immune-stimulatory signals

The most prominent pattern was a widespread downregulation of genes involved in immune activation and surveillance. A total of 19 genes showed decline in expression, suggesting a progressive erosion of the immunological landscape within the tumor microenvironment. This included key chemokines such as XCL1, which mediates dendritic cell-driven cytotoxic responses, and CCL24, CCL14, and CCL25, whose reduced expression limits leukocyte recruitment. Similarly, chemokines receptors CCR3 and CXCR1 were downregulated, potentially impairing immune cell trafficking. Inflammatory cytokines including IL17A, IL15, and IL21 also showed marked reduction in late-stage tumors, consistent with the role of IL-15 in promoting antitumor responses.^51^ Importantly, B2M, which encodes β2-microglobulin essential for MHC-I antigen presentation, was downregulated, likely contributing to immune evasion, as described in multiple cancers.^52^ Overall, this transcriptional repression points to a collapse of adaptive immune signaling in metastatic PDAC.

#### Selective upregulation of immunoregulatory signals

Conversely, 6 genes were upregulated at advanced stages, chemokines CCL23 and CXCL16, which are associated with angiogenesis and recruitment of immunosuppressive cells like tumor-associated macrophages and regulatory T cells. CCR4, involved in the differentiation of Th2 and Treg cells, also showed increased expression. Surprisingly, IL-18 was upregulated despite the general decline in proinflammatory signals. Since IL-18 requires caspase-1 mediated cleavage to become active, its elevated transcript levels may reflect accumulation of the inactive precursor, insufficient to mount an effective immune response. The lack of adaptive immune signaling in metastatic PDAC may reflect that IL-18 persist as inactive being inefficient in inducing an effective antitumor immune response.

Collectively, these findings reinforce the paradigm of PDAC as an “immune-cold” tumor. The transcriptomic data highlights an active remodeling of the immune microenvironment characterized by suppressed antigen presentation (e.g., MHC-I via B2M), diminished effector cytokine expression (e.g., IL17A, IL15, and IL21), reduced immune infiltration (e.g., CCR3 and CXCR1), and enhanced immunosuppressive signals (e.g., CCR4 and CXCL16). These adaptations may be driven by tumor-intrinsic mechanisms such as autophagy, which has been shown to degrade MHC-I in PDAC cells.^53^

### Comprehensive deconvolution and regulatory landscape of the stromal microenvironment during PDAC progression

**Figure 6:**
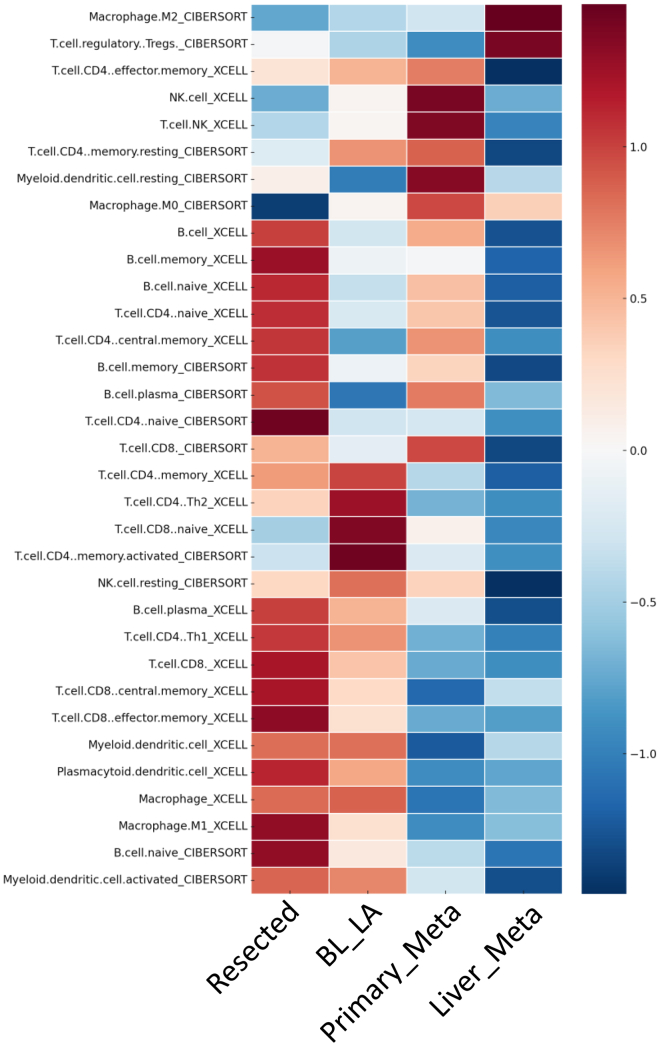
Heatmap showing the relative abundance of immune cell types across different disease stages The figure presents immune cell infiltration levels estimated using the CIBERSORT and xCELL algorithms. Each row represents a distinct immune cell type, and the columns correspond to disease stages. The color intensity reflects the relative abundance of each cell type: red indicates higher abundance, while blue indicates lower abundance. White represents intermediate levels. Cell types inferred by CIBERSORT and xCELL are labeled on the vertical axis.

We employed the xCELL and CIBERSORT algorithms to computationally infer and quantify a broad spectrum of immune and stromal cell types within the PDAC tumor microenvironment. The heatmap (Figure 6) displays the relative abundance (*Z* score normalized) of key cell populations, including distinct subsets of B cells (naive, memory, and plasma), T cells (CD4^+^ and CD8^+^ subsets including regulatory, naive, Th1, Th2, central memory, and effector memory), natural killer (NK) cells (resting and xCELL-inferred NK types), macrophages (M0, M1, and M2), myeloid and plasmacytoid dendritic cells, as well as cancer-associated fibroblasts (CAFs) and other stromal components. This high-resolution deconvolution revealed distinct enrichment or depletion patterns across disease stages and sample groups. In addition to cell-type abundance, we calculated immune, stromal, and composite microenvironment scores to further characterize tumor immune-interactions. Correlation analyses between cell-type frequencies and key regulator factors (e.g., cytokines, transcription factors, and ECM-related pathways) uncovered cell state-specific molecular programs. Elevated CAF levels, for example, were associated with fibroblast-activating signals, while macrophage polarization aligned with pro-inflammatory cytokine expression. Overall, this integrative approach defines the complex regulatory landscape of the PDAC stroma and identifies key cell-type-specific drivers of microenvironmental heterogeneity.

Transcriptomic and immune deconvolution analyses revealed a profound, stage-dependent remodeling of the tumor immune microenvironment (TME) throughout PDAC progression. In resectable tumors, the TME displayed an immune-active phenotype, enriched in cytotoxic CD8^+^ T cells, dendritic cells, and M1 macrophages, together with elevated expression of pro-inflammatory cytokines such as IL15, IL17A, and IL21, and preserved B2M levels sustaining MHC-I-mediated antigen presentation. This configuration reflects an immune-competent context able to sustain partial antitumor surveillance. However, during progression to borderline/locally advanced and metastatic stages, there is a gradual and coordinated suppression of immune activation programs. Genes mediating effector cell recruitment and activation are progressively silenced, while immunosuppressive mediators (CCR4 and CXCL16) become upregulated, promoting Treg and M2 macrophage polarization. Concomitantly, a decline in CD8^+^ T and NK cell infiltration, together with enrichment of fibroblasts and endothelial elements, indicates stromal expansion and immune exclusion. In liver metastases, this process culminates in a fully “immune-cold” state marked by the loss of antigen presentation machinery (B2M), diminished cytokine signaling, and dominance of stromal and myeloid suppressive components. Overall, these findings delineate a dynamic continuum from an immune-inflamed to an immune-suppressed TME, characterized by loss of effector immunity, emergence of tolerogenic stroma, and metabolic adaptation favoring tumor persistence and dissemination.

### Integrated gene expression and immune remodeling during PDAC progression

**Figure 7:**
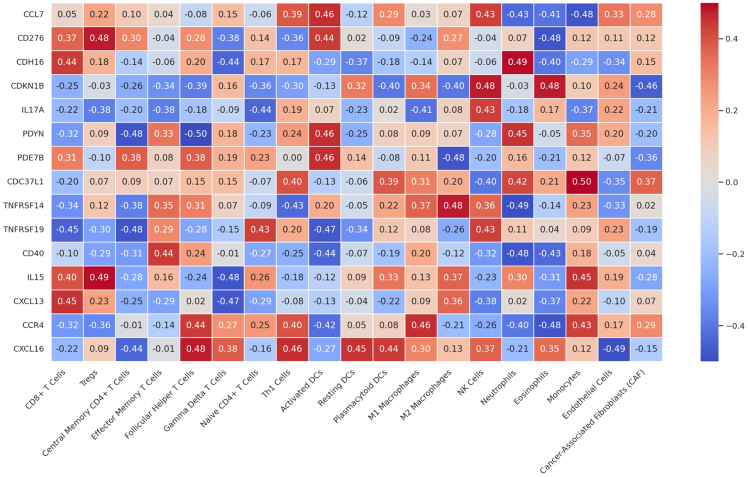
Correlation heatmap between tumor-derived factors and immune/stromal cell populations in the PDAC microenvironment Heatmap displaying statistically significant correlations (*p* < 0.05) between the expression of selected tumor-associated factors (*x* axis) and the inferred abundance of immune and stromal cell types (*y* axis) in the PDAC microenvironment. Cell types were estimated by computational deconvolution of bulk transcriptomic data using CIBERSORT and xCell algorithms. The color scale represents the Pearson correlation coefficient (*Z* score standardized), with positive correlations shown in red and negative in blue. Asterisks indicate statistical significance levels: *p* < 0.05 (∗), <0.01 (∗∗), and <0.001 (∗∗∗). Notable observations include: Positive associations of IL1A, PDGFB, TNFRSF9, and TNFSF4 with macrophages (M2-like and general), dendritic cells, and fibroblast populations, suggesting a potential role in supporting stromal remodeling and immunosuppressive niches. Negative correlations of CD276 and IL13RA1 with T cell subsets (e.g., CD4^+^ and CD8^+^ naive, central memory), implying potential immune evasion mechanisms. Stromal and immune scores derived from xCell show strong associations with PDGFRB, POU, and IL1A, reinforcing their involvement in shaping the tumor microenvironment. This analysis highlights specific molecular mediators potentially responsible for immune modulation and stromal activation during PDAC progression, offering insights into tumor-microenvironment interactions and candidate targets for immunomodulatory therapy.

To investigate the immunological alterations accompanying PDAC progression, we conducted a transcriptome-wide analysis across disease stages. A progressive decline in global gene expression was observed from resected to liver_meta samples (Figure 6), suggesting widespread transcriptional suppression. This downregulation likely reflects loss of epithelial function, reduced metabolic activity, and impaired immune surveillance, hallmarks of cancer progression and immune escape.^54^^,^^55^ We next explored associations between gene expression and immune cell-type abundance (Figure 7). Pearson correlation coefficients (r) were computed between 15 candidate genes and immune signatures. Associations were considered biologically meaningful if statistically significant (*p* < 0.05) and at least moderately strong (r ≥ 0.3). Among the tested genes, IL15, TNFRSF14 (HVEM), and CXCL16 showed the most consistent positive correlations, reaching values of r ≈ 0.40–0.49, linking them to effector and antigen-presenting immune populations. PDYN displayed a mixed profile, including a strong positive association (r ≈ 0.46) but also several negative correlations (down to r ≈ −0.25), suggesting a context-dependent role in immune regulation. CDC37L1 also showed at least one moderate positive correlation (r ≈ 0.40). In contrast, IL17A exhibited predominantly negative and low-magnitude correlations (minimum r ≈ −0.23; maximum positive only r ≈ 0.08), contradicting its expected pro-inflammatory profile. CDKN1B, TNFRSF19, and CXCL13 showed weak but generally inverse correlations (down to r ≈ −0.25/-0.26), whereas CCR4 and CD40 correlated only modestly with immune subsets (maximum r ≈ 0.25–0.26).

### Metabolic flux inference and pathway activity across clusters

Although our analysis is based primarily on unsupervised clustering and differential gene expression profiling, we complemented these approaches with functional pathway analyses and metabolic flux inference to enhance biological interpretation. Integrating these data with survival outcomes and independent validation cohorts reinforces the translational relevance of the identified programs and suggests that stage-specific transcriptional shifts are mechanistically linked to metabolic adaptation, immune remodeling, and therapeutic vulnerability in PDAC. To further explore inter- and intra-patient variability, we performed a patient-wise expression analysis of the key molecular programs identified in our cluster-level analyses. Specifically, we examined the normalized expression of representative genes associated with immune activity (IL15, B2M, and CXCL16), metabolic adaptation (RAC3, COQ7, and EPAS1), and cellular plasticity (ZEB1, SNAI2, and MYC) across individual tumor samples and disease stages. This analysis reveals substantial inter-patient heterogeneity, particularly within the intermediate BL/LA and primary metastasis stages. Despite this variability, the data support a model of convergent transcriptional trajectories driving PDAC progression across diverse patient contexts. While our integrative analysis highlights potential therapeutic targets and stage-specific molecular vulnerabilities, these findings should be considered hypothesis-generating rather than confirmatory. Functional and pharmacologic validation will be required to determine whether these pathways represent actionable dependencies in PDAC. To explore metabolic pathway dynamics in greater detail, we employed in silico metabolic flux activity analysis based on the RECON2 human metabolic network. Using ssGSEA-derived pathway activity scores, we inferred stage- and cluster-specific metabolic reprogramming patterns. Early-stage clusters (C2-C4) exhibited enrichment of glycolytic, pentose phosphate, and nucleotide biosynthetic pathways, consistent with anabolic metabolism supporting proliferation. In contrast, late-stage clusters (C6-C8) showed increased flux through oxidative phosphorylation (OXPHOS), fatty acid β-oxidation, and amino acid degradation, reflecting enhanced mitochondrial activity and metabolic plasticity. These metabolic shifts paralleled transcriptional activation of key enzymes including COQ7, EPAS1, NUAK1, and RAC3, and were strongly associated with metastatic competence. Together, these results suggest that PDAC progression involves a coordinated transition from glycolytic to oxidative metabolic programs, supporting energy flexibility and survival during dissemination. In addition, these results highlight IL15, TNFRSF14, and CXCL16 as the strongest immune-associated transcripts, reinforcing their links to pro-inflammatory signaling and immune activation. Conversely, IL17A and several other genes demonstrated weaker or inconsistent associations, underscoring the heterogeneity of immune regulation in PDAC and pointing to potential context-dependent or immune-suppressive mechanisms.

### Therapeutic implications and stage-dependent drug sensitivity

Integration of transcriptional programs with pharmacogenomic databases suggests that PDAC progression is accompanied by shifting therapeutic vulnerabilities. Early-stage clusters, characterized by preserved epithelial identity, immune activation, and glycolytic metabolism, exhibit expression profiles compatible with sensitivity to nucleoside analogs and DNA-damaging agents, such as gemcitabine and fluorouracil. This is supported by the relative upregulation of genes involved in nucleotide transport and replication stress (RRM1/2, MCM family, and PCNA), which increase reliance on DNA synthesis pathways targeted by these drugs. Conversely, late-stage and metastatic clusters display strong activation of oxidative phosphorylation, redox control, and PI3K-AKT-mTOR signaling (EPAS1, COQ7, RAC3, and NUAK1), suggesting preferential sensitivity to metabolic inhibitors (e.g., OXPHOS or fatty acid oxidation inhibitors) and pathway-targeted agents such as mTOR or PI3K inhibitors. The concomitant loss of immune surveillance and enrichment of immunosuppressive cues (CCR4 and CXCL16) also supports potential benefit from immune checkpoint blockade or Treg-targeted strategies when combined with metabolic interference. Together, these associations highlight that early PDAC stages may respond more favorably to cytotoxic and nucleoside-based chemotherapy, while advanced or metastatic disease may require combination approaches integrating metabolic and immune modulation to overcome resistance and immune evasion. Together, these findings delineate stage-specific transcriptional programs that shape PDAC progression and reveal molecular pathways with potential therapeutic relevance. Although the present study does not include experimental validation, the identified genes and pathways (RAC3, COQ7, EPAS1, CCR4, and CXCL16) provide a testable framework for future studies aimed at evaluating their causal roles and therapeutic tractability. Thus, our results should be interpreted as hypothesis-generating rather than confirmatory, offering a systems-level foundation for identifying and prioritizing candidate targets in PDAC.

### Interpatient heterogeneity of critical transcriptional programs

To evaluate the degree of interpatient variability, we examined the expression patterns of the top progression-associated genes (CDH12, FHIT, SLC14A2, RAC3, EPAS1, COQ7, and LOXL1) across individual samples. The results demonstrate marked heterogeneity within each clinical stage, with some early-stage tumors already exhibiting partial activation of metastatic-associated programs, and conversely, a subset of advanced cases retaining epithelial or immune-active signatures. These findings underscore the high degree of transcriptional heterogeneity in PDAC and suggest that disease progression follows a continuum rather than discrete molecular states.

### Early- and late-stage gene signatures and external validation

To further improve clinical translatability, we derived two minimal gene sets that summarize the early and late transcriptional states observed in our stage-resolved map. The early-stage signature, composed of CDH12, SLC14A2, FHIT, and IL17RD, is highly expressed in Cluster C1 and progressively downregulated from resected to liver metastatic PDAC. Conversely, the late-stage signature, comprising RAC3, EPAS1, COQ7, and LOXL1, is preferentially activated in cluster C2 and enriched in primary metastatic and liver metastasis samples. In the discovery cohort, higher early-scores were associated with lower clinical stage and more favorable survival, whereas higher late-scores tracked with advanced stage and poorer outcome. Importantly, these patterns were recapitulated in independent RNA-seq datasets from TCGA-PAAD and ICGC PACA-AU, where the early- and late-scores showed consistent, stage-aligned trends and stratified OS in the expected direction. Together, these findings support the existence of compact, biologically grounded gene signatures that capture the continuum from an epithelial/homeostatic to a metastatic/metabolically adapted state, and highlight their potential utility as clinically deployable markers for PDAC stratification.

### Functional validation of transcriptome-based hypotheses in patient-derived PDAC cultures

To functionally validate hypotheses derived from transcriptomic analyses, we performed *in vitro* assays using patient-derived PDAC cultures representing distinct stages of disease progression: five from resected primary tumors, five from primary tumors of patients with metastatic disease (primary_meta), and four from liver metastases (liver_meta) previously reported.^56^^,^^57^ This design enabled direct evaluation of stage-associated phenotypes linked to invasion, redox regulation, and metabolic adaptation.

**Figure 8:**
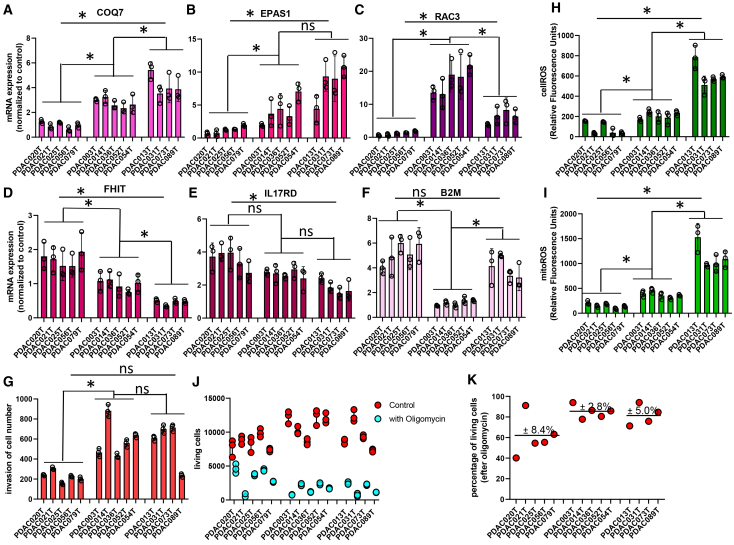
Functional validation of transcriptome-derived progression programs in patient-derived PDAC cultures Patient-derived PDAC primary cultures were grouped as localized primary tumors (resected, *n* = 5), primary tumors from patients with metastatic disease (primary, *n* = 5), and liver metastasis-derived cultures (liver_meta, *n* = 4). (A–F) Relative mRNA expression of six selected genes measured by RT-qPCR and expressed as fold-change, relative to the housekeeping gene. Three transcripts previously identified as upregulated during progression and three as downregulated are shown, confirming stage-associated expression patterns in patient-derived cultures. (G) Invasive capacity assessed by Matrigel invasion assay and expressed as number of invading cells. Cultures derived from metastatic and liver metastasis samples display increased invasive activity compared with localized tumor-derived cultures. (H and I) Basal total reactive oxygen species (ROS) levels measured using CellROX green and basal mitochondrial ROS levels measured using MitoSOX Red and quantified as mean fluorescence intensity (MFI, arbitrary units) by flow cytometry. Substantial inter-sample heterogeneity is observed, with higher ROS levels generally detected in advanced-stage cultures. Mitochondrial ROS levels vary among samples and do not strictly correlate with total ROS, suggesting contribution of non-mitochondrial ROS sources in a subset of tumors. (J) Paired comparison of control versus oligomycin-treated conditions for each individual culture, showing mean of technical triplicates per sample and highlighting inter-sample variability in mitochondrial dependency. (K) Percentage reduction in viable cells after oligomycin treatment calculated relative to matched controls for each individual culture. Grouped analysis of oligomycin sensitivity expressed as mean percentage reduction ±SEM. Resected samples cultures show lower sensitivity (60.9 ± 8.4%) compared with primary (84.9 ± 2.8%) and liver_meta (81.4 ± 5.0%), indicating increased dependence on mitochondrial oxidative phosphorylation in advanced-stage PDAC cultures. Statistical analyses were performed using non-parametric tests with Dunn’s post hoc multiple-comparison correction; significance levels are indicated in the figure (∗*p* < 0.05; ns, not significant).

We first assessed the expression of six mRNAs selected from the transcriptomic dataset, including three genes that increase (COQ7, EPAS1, and RAC3) and three that decrease (FHIT, IL17RD, and B2M) with tumor progression. Quantitative RT-qPCR confirmed expression patterns consistent with the RNA-seq data, with progressive and metastatic-derived cultures showing higher expression of late-stage markers and lower expression of early-stage markers (Figures 8A–8F). These results indicate that key progression-associated transcriptional programs are preserved in patient-derived cultures.

To determine whether activation of cytoskeletal and migration pathways translates into functional changes, we measured invasive capacity using Matrigel invasion assays. Cultures derived from metastatic tumors and liver metastases exhibited significantly higher invasive activity than cultures from localized tumors (Figure 8G), supporting a functional link between progression-associated gene expression programs and invasive behavior.

Basal levels of total reactive oxygen species (ROS), measured using CellROX Green, varied substantially across PDAC cultures, indicating intrinsic heterogeneity in redox status (Figure 8H). Mitochondrial ROS levels, assessed by MitoSOX Red, also showed marked inter-sample variability (Figure 8I). Notably, elevated total ROS did not always correspond to proportionally increased mitochondrial ROS, suggesting that non-mitochondrial sources contribute to oxidative stress in a subset of tumors. Overall, progressive and metastatic-derived cultures tended to display higher total and/or mitochondrial ROS levels, consistent with increased metabolic activity during tumor progression.

To evaluate mitochondrial dependency, cultures were treated with the ATP synthase inhibitor oligomycin for 24 h, and viable cell numbers were quantified relative to vehicle-treated controls. Oligomycin significantly reduced viable cell numbers across all samples, indicating a general contribution of mitochondrial function to short-term survival (Figure 8J). However, the magnitude of the response varied among cultures, revealing heterogeneous sensitivity to OXPHOS inhibition. Quantification of percentage reduction in viable cells showed that cultures from localized tumors exhibited lower sensitivity (60.9 ± 8.4%) compared with cultures from primary_meta tumors (84.9 ± 2.8%) and liver_meta (81.4 ± 5.0%) (Figure 8K), indicating increased dependence on mitochondrial oxidative phosphorylation in advanced-stage PDAC cultures.

Together, these functional assays demonstrate that PDAC progression is associated with increased invasiveness, altered redox balance, and enhanced reliance on mitochondrial metabolism, while also revealing substantial inter-sample heterogeneity. The partial dissociation between total ROS and mitochondrial ROS in some samples suggests that oxidative stress is not uniformly driven by mitochondrial respiration, supporting the presence of distinct metabolic programs across tumors. These findings provide functional support for transcriptome-derived predictions of metabolic rewiring during PDAC progression and identify mitochondrial dependency as a stage-associated vulnerability in a subset of PDAC tumors.

### Concluding remarks

Deciphering the molecular processes driving tumor transformation and progression is crucial to understanding cancer complexity. In this study, we dissected these stages and uncovered key mechanisms underlying PDAC development. This knowledge is not only fundamental to comprehending the disease at its core, but it also plays a pivotal role in guiding the design of more precise and effective therapies. A complete understanding of PDAC progression requires appropriate models and human samples. Human tissue allows for the study of distinct stages of tumor evolution, while genetically engineered mouse models and cell-based systems are necessary to explore early transformation events under controlled conditions. Our findings highlight specific immune and metabolic adaptations that occur during PDAC progression and that hold strong clinical relevance. Immune-targeted therapies could involve enhancing pro-inflammatory responses, particularly via CD8^+^ T cells and M1 macrophages, while inhibiting suppressive Tregs and M2 macrophages. The detection of residual immune activity offers hope for the development of novel immunotherapeutic strategies. Likewise, targeting metabolic dependencies, such as glycolysis, lipid metabolism, or oxidative phosphorylation, may be particularly effective in advanced and metastatic stages. Monitoring stromal and immune markers can also provide insight into disease dynamics and help guide personalized treatment. Through our research, we have uncovered that each stage of PDAC progression involves the activation of distinct molecular pathways, many of which had been previously underappreciated. These pathways offer novel, stage-specific therapeutic targets. A tailored, progression-aware approach may enable more precise and effective interventions for this highly aggressive disease.^58^^,^^59^^,^^60^ Our analyses were primarily grounded in unsupervised clustering and differential gene expression profiling, complemented by integrative functional pathway interrogation and metabolic flux inference to refine biological interpretation. Coupling these multidimensional datasets with survival outcomes and independent validation cohorts underscores the translational robustness of the identified molecular programs. Collectively, the data implicate stage-dependent transcriptional reprogramming in PDAC as a mechanistic nexus linking metabolic adaptation, immune landscape remodeling, and context-specific therapeutic vulnerabilities. Overall, functional analyses show that PDAC progression involves increased invasiveness, redox imbalance, and enhanced reliance on mitochondrial metabolism, with substantial inter-sample heterogeneity. The partial dissociation between total and mitochondrial ROS suggests distinct metabolic programs across tumors, supporting mitochondrial dependency as a stage-associated vulnerability in a subset of PDAC cases.

In summary, PDAC progression involves a complex interplay of immunosuppression, metabolic reprogramming, and microenvironmental remodeling. While these features contribute to tumor aggressiveness, they also represent actionable vulnerabilities. Combining metabolic inhibitors, immune-modulating agents, and strategies targeting the tumor microenvironment may help overcome resistance and improve outcomes in PDAC patients. These findings highlight putative stage-specific vulnerabilities that warrant functional validation in preclinical models.

Limitations: this study has several limitations. First, the analyses are based on bulk RNA-seq data from FFPE tissue without systematic microdissection, which may introduce variability due to cellular admixture and desmoplasia. We estimated tumor purity and repeated key analyses adjusted for stromal and immune content, but residual confounding cannot be excluded. Second, immune remodeling across PDAC stages was inferred mainly through computational deconvolution (xCell, CIBERSORT, and ESTIMATE), complemented by single-cell meta-analysis for directional context. Although these methods enable large-scale inference, orthogonal validation through multiplex immunohistochemistry, and spatial transcriptomics remains ongoing. Third, our results are primarily correlative and do not establish causality. Functional experiments are underway to test the roles of selected candidates (RAC3, COQ7, EPAS1, ZEB1, and IL15) in invasion, redox regulation, and immune modulation using CRISPR interference, gain-of-function assays, and organoid and mouse models. Fourth, while an external validation confirmed the robustness of a compact gene panel, cohort heterogeneity and platform differences may limit generalizability; thus, prospective validation is warranted. Finally, the drug-response analyses are exploratory and should be viewed as hypothesis-generating rather than clinically predictive. Despite these limitations, our integrated analyses provide a systems-level view of PDAC progression. The convergence of oncogenic, metabolic, and immune remodeling programs across disease stages suggests a coherent mechanistic framework. Future studies using single-cell and spatial transcriptomic approaches will be essential to dissect the cellular crosstalk underlying these transcriptional programs and to validate their causal and therapeutic relevance.

## Resource availability

### Lead contact

Further information and requests for resources, code, and data should be directed to and will be fulfilled by the lead contact, Juan Iovanna (juan.iovanna@inserm.fr).

### Materials availability

This study did not generate new unique reagents.

### Data and code availability

• The RNA-sequencing data used in this study are available at https://doi.org/10.5281/zenodo.18619250.
• Data: The data that support the findings of this study are available from the corresponding author upon reasonable request.
• Code: All analysis code (R scripts and Rmarkdown notebooks) and locked package versions are available at a public Git repository (URL provided upon acceptance).
• Additional information: Any remaining information required to reanalyze the data reported in this paper is available from the lead contact upon request.
• The RNA-sequencing data used in this study were obtained from four publicly available or previously published cohorts (GemPred, GemPred Retro, Massachusetts General Hospital, and Hospital de Clínicas “José de San Martín” (https://doi.org/10.5281/zenodo.18619250). Data processing and analytical pipelines are described in the STAR Methods section. Processed gene-expression matrices and associated metadata are available from the corresponding authors upon reasonable request.

## Acknowledgments

This work was supported by 10.13039/501100014087INCa (grants 2018-078 to N.D. and 2018-079 to J.I.), 10.13039/501100006331Canceropole PACA (to N.D.), 10.13039/501100001677INSERM (to J.I.), 10.13039/501100003074ANPCyT (PICT 2021 GRF-TII-00050 to D.G.; PICT 2020 Serie A-02624 to M.N.G.), and 10.13039/501100002923CONICET (PIP-2020-11220200102518CO to D.G.).

## Author contributions

Conceptualization, resources, data curation, validation, investigation, methodology, writing – review & editing, E.C.; formal analysis, B.C.; formal analysis, A.M.; formal analysis, validation, investigation C.R., A.P.d.L., K.M., C.I., D.G., G.K., F.G., M.L., J.G., C.D., A.Y., A.S., D.G., M.N.G., D.P., M.E.P., L.S., F.G.L., J.G., A.P., and O.M.; conceptualization, resources, formal analysis, validation, investigation, P.H.; formal analysis, supervision, writing – original draft, writing – review & editing, N.F.; conceptualization, formal analysis, supervision, writing – original draft, writing – review & editing N.D.; conceptualization, data curation, formal analysis, supervision, funding acquisition, project administration, writing – original draft, writing – review & editing, J.I.

## Declaration of interests

The authors declare no potential conflicts of interest.

## Declaration of generative AI and AI-assisted technologies in the writing process

During the preparation of this work, the authors used ChatGPT in order to in order to assist with drafting and improving the clarity of the text. After using this tool, the authors reviewed and edited the content as needed and take full responsibility for the content of the publication.

## STAR★Methods

### Key resources table

**Table:** 

REAGENT or RESOURCE	SOURCE	IDENTIFIER
**Chemicals, peptides, and recombinant proteins**		

Collagenase type V	Sigma-Aldrich	C9263
DMEM/F12 + supplements	Multiple suppliers	SFDM conditions
36B4	This study	ΔCt normalization
Transwell inserts 8 μm	Corning	Cat#353097
Matrigel (1:5)	Corning	N/A
Mitomycin C	Sigma-Aldrich	10 μg/mL
Oligomycin	Sigma-Aldrich	2 μM, 24 h

**Critical commercial assays**		

AllPrep FFPE Kit	Qiagen	DV200 RNA quality metric
QuantSeq 3’ mRNA-Seq Kit	Lexogen	3’ RNA-seq
RNeasy kit	Qiagen	N/A
GoScript RT System	Promega	N/A
AriaMx	Agilent	N/A
CellROX Green	Thermo Fisher	C10444
MitoSOX Red	Thermo Fisher	M36008
CytoFLEX S	Beckman Coulter	≥10,000 cells/sample

**Experimental models: Cell lines**		

PDAC PDX-derived cultures	PaCaOmics trial	NCT01692873

**Software and algorithms**		

GRCh38 reference genome	Genome Reference Consortium	Human genome build
Rsubread	Bioconductor	R package
TMM normalization	edgeR	log2-CPM
ComBat	sva package	Batch = cohort; covariate = stage
limma-voom	Bioconductor	FDR <0.01; |log2FC|>1
Mfuzz soft clustering	R Mfuzz package	m=1.25; membership>0.7
GO, KEGG, Reactome	clusterProfiler v4.8.3	Pathway analysis
GSVA, ssGSEA	GSVA package	Scoring methods
xCell	Aran et al.^61^	Enrichment scores
CIBERSORT (LM22)	Newman et al.^62^	Relative fractions
ESTIMATE	Yoshihara et al.^63^	Immune/Stromal scores
RECON2 gene sets	Human metabolic network	Transcript inference
LASSO regression	glmnet v4.1	10-fold CV
TCGA-PAAD RNA-seq	TCGA	Public dataset
ICGC PACA-AU	ICGC	Public dataset
Kaplan–Meier, Cox, non-parametric tests	R	Adjusted p<0.05
ggplot2, ComplexHeatmap	R packages	N/A

### Experimental model and study participant details

#### Human subjects

We analyzed **443 treatment-naïve PDAC** tumors obtained from institutional and collaborating biobanks. Samples were classified into **Resected, Borderline/Locally Advanced (BL/LA), Primary Metastatic**, and **Liver Metastasis** groups according to clinicopathologic records. Inclusion criteria: histologically confirmed PDAC, treatment-naïve status, adequate FFPE tissue for RNA extraction, and available minimal clinical data (age, sex, stage, survival/follow-up). Samples with <70% tumor cellularity were not excluded but were subsequently **adjusted for purity** in the analyses. The study complied with the Declaration of Helsinki and was approved by local IRBs/ethics committees; all specimens were obtained with informed consent or under approved waiver.

#### Ethics approval

Human tumor samples were obtained from four independent cohorts: the GemPred,^64^ GemPred Retro,^65^ Massachusetts General Hospital,^66^ and Hospital de Clínicas “José de San Martín” cohorts. The Hospital de Clínicas cohort was approved by the local Ethics Committee of the Hospital de Clínicas “José de San Martín,” Universidad de Buenos Aires, Argentina. All samples were collected from treatment-naïve patients in accordance with institutional and national ethical guidelines.

### Method details

#### RNA extraction, sequencing, and initial QC

FFPE blocks were macro-dissected to enrich tumor content when feasible. RNA was extracted using standard kits compatible with FFPE material, quality checked (RIN/DV200), and sequenced as stranded, poly(A)-selected (or rRNA-depleted when poly(A) was not feasible) libraries on Illumina platforms (paired-end 75–150 bp). FASTQ QC used **FastQC v0.11.9** and **MultiQC v1.14**.

#### Read alignment and quantification

Reads were aligned to **GRCh38** using **STAR v2.7.11a** with default settings plus twopassMode Basic. Gene-level counts were obtained via **featureCounts v2.0.3** (Gencode v38 annotation). Alternative quantification for sensitivity analyses used **Salmon v1.10** (quasi-mapping; TPMs). Only **protein-coding genes** and **annotated lncRNAs** with **CPM > 1 in ≥20%** of samples were retained.

#### Normalization and batch correction

Raw counts were **TMM-normalized (edgeR v3.44.0).** Log2-CPM were computed with precision weights. To mitigate inter-cohort effects, we applied **ComBat** (**sva v3.48.0;** parametric; batch = acquisition cohort; covariates = clinical stage, age, sex) after verifying the presence of batch structure by PCA and kBET.

#### Differential expression and effect sizes

Primary comparisons across stages were performed with **limma-voom v3.58.1** using linear models with empirical Bayes moderation. Unless stated otherwise, we report genes with **FDR (Benjamini-Hochberg) < 0.05** and **|log2FC| ≥ 0.5** as differentially expressed.

#### Dynamic program inference across progression

To capture stage-dependent trends, we used **Mfuzz v2.60.0** on z-scored, batch-corrected expression for genes variable across samples (top 5,000 by MAD). Fuzzifier m was selected by mestimate (typical range 1.2–1.6), number of clusters determined by Dunn/MC indices and inspection of cluster stability (bootstrap, 1,000 resamples). We retained **10 dynamic programs** (C1-C10) showing coherent monotonic or biphasic patterns across stages.

#### Pathway enrichment (GSEA/GO/KEGG/Reactome)

We performed **GSEA (fgsea v1.28.0)** using preranked gene lists (t-statistic from limma) against **MSigDB v7.5.1** Hallmark, Reactome, KEGG, and GO Biological Process. For each program and stage contrast, we report **NES, padj** (BH), and leading-edge genes. Enrichment was considered significant at **padj < 0.05**.

#### Sample-level pathway activity (GSVA/ssGSEA)

We computed per-sample activities using **GSVA v1.50.0** with Gaussian kernel and **ssGSEA** for selected signatures (glycolysis, OXPHOS, lipid metabolism, hypoxia, EMT, interferon signaling). Scores were compared across stages by **ANCOVA** adjusting for purity (see below) and multiple testing (BH).

#### Immune/stromal deconvolution

We estimated relative cell-type enrichment using **xCell (webtool/v1.1 signatures)** and **CIBERSORT (LM22)** on TPM-scaled matrices. For each sample we computed CD8 T cell, Treg, M1/M2 macrophage, dendritic cell, NK, fibroblast, and endothelial scores. We also computed **ESTIMATE v1.0.13** Immune, Stromal, and Purity scores. Group comparisons used linear models with purity and batch as covariates. We present boxplots, heatmaps, and correlation matrices relating sentinel genes (e.g., IL15, B2M, CCR4, CXCL16) to immune estimates.

#### Tumor purity estimation and adjustment

Primary purity estimation used **ESTIMATE Purity** (transformed from Immune/Stromal). As sensitivity analyses, we used **DeconRNASeq v1.46.0** with epithelial/stromal/immune reference signatures. All key differential analyses were (i) **adjusted** for purity as a covariate and (ii) **stratified** by purity tertiles to test robustness. We report any discrepancies in Supplementary Figures/Tables.

#### Survival analyses

OS was modeled using **Cox proportional hazards** (R **survival v3.5-7**). Proportionality was verified via Schoenfeld residuals; violations were handled by time-varying coefficients or stratification. Kaplan-Meier curves were compared with log rank tests. Because follow-up varied, we additionally report **restricted mean survival time (RMST)** at 36 and 60 months (**survRM2 v1.0-5**). Multivariable models included age, sex, clinical stage, and **purity** (ESTIMATE) as covariates.

#### Compact gene panel (feature selection, modeling, validation)

We derived a minimal gene set for stage classification and/or risk stratification.

1 **Feature preselection:** intersection of (a) DE genes with **FDR < 0.01** and **|log2FC| ≥ 0.7** across progression-relevant contrasts; (b) members of dynamic programs with high membership (Mfuzz membership ≥0.7); (c) biology-anchored genes from leading edges (GSEA).
2 **Modeling: Elastic-Net/LASSO** using **glmnet v4.1-8** (10-fold CV, 100 lambda values; alpha tuned 0.2–1.0). Classification performance reported as **AUC** (bootstrap 1,000 iterations), calibration (Hosmer-Lemeshow ), and **decision-curve analysis** (**rmda v1.6**). For survival, penalized **Cox** with time-dependent AUC (**timeROC v0.4**).
3 **External validation:** Locked models were validated in **TCGA-PAAD** and **ICGC PACA-AU** (treatment-naïve subsets; harmonized TPM, ComBat on “dataset” with stage as covariate). We report external **AUC**, calibration, and KM curves (dichotomized by median score), plus HRs with 95% CIs.

#### Candidate-gene short list (the “15 genes”) and transparency

The 15 candidates shown in the main figure were selected by a **pre-specified multi-criterion rule**.

✓ present in at least one **leading edge** from GSEA for a progression contrast;
✓ **monotonic trend** across stages (Jonckheere-Terpstra **p < 0.01**);
✓ **DE** with **FDR < 0.01** and **|log2FC| ≥ 0.7** in ≥2 adjacent-stage contrasts;
✓ **prognostic association** (Cox **q < 0.1**) consistent in discovery and directionally concordant in external cohorts;
✓ robust to purity adjustment;
✓ biologically interpretable within immune/metabolic/cytoskeletal axes.
✓ A sensitivity analysis shows stability of the list to threshold perturbations (±20%).

#### Drug-response inference (exploratory)

We estimated putative drug sensitivity using **oncoPredict v1.2** and **pRRophetic v0.5** trained on **GDSC/PRISM** pharmacogenomic data. Stage/cluster signatures were projected to predict **IC50-like** metrics for gemcitabine, FOLFIRINOX components, PI3K/mTOR inhibitors, and OXPHOS modulators. We emphasize these results are **hypothesis-generating** only.

#### Single-cell concordance meta-analysis (directional check)

To contextualize bulk findings, we compared stage-wise trends to **public PDAC scRNA-seq** references. Cell-type proportion shifts (e.g., CD8 T cells, Tregs, macrophage states) were summarized qualitatively across datasets, focusing on **directional concordance** with our deconvolution. Where available, we projected our signatures onto scRNA-seq epithelial clusters using **AUCell v1.22.0**.

#### Heterogeneity analyses

We quantified **inter-patient variability** by stage and program using variance components and **Levene’s tests**. Patient-wise distributions for key markers were plotted as **waterfall** and **violin** plots. **PCA/UMAP** (Seurat v4.4 on scaled bulk expression) visualized separation by stage/cluster and the dispersion within groups.

#### Cohorts sequencing

A total of 443 samples from PDAC patients were included in the study cohort. These included 35 samples localized, 244 borderline or locally advanced (BL_LA), and 164 metastatic cases (76 primary pancreatic and 88 liver metastasis). All samples were treatment-naïve. Samples were sourced from four independent cohorts: 215 from the GemPred cohort,^64^ 43 from Massachusetts General Hospital,^66^ 21 from the Hospital de Clínicas “José de San Martín” cohort (approved by the ethical committee of the Hospital de Clínicas “José de San Martín”), and 164 from the GemPred Retro cohort.^65^ Only these cohorts were included due to the availability of sufficient high-quality material for RNA extraction. RNA was isolated from FFPE blocks using the AllPrep FFPE Kit (Qiagen, Hilden, Germany) and RNA quality was assessed based on the DV200 metric (percentage of fragments >200 nucleotides). RNA libraries were prepared using the QuantSeq 3′ mRNA-seq Kit (Lexogen, Vienna, Austria).

#### RNA sequencing and analysis

Sequencing reads were aligned to the human reference genome (GRCh38) using the Rsubread package in R,^67^ and gene-level read counts were generated. Genes with low expression were filtered by retaining those with counts per million (CPM) > 1 in at least 10% of samples. Library sizes were normalized using the trimmed mean of M-values (TMM) method implemented in edgeR, and normalized counts were transformed to log2-CPM values for downstream analyses. To correct for technical variability across cohorts, batch effects were adjusted using the ComBat function from the sva package,^68^ with cohort included as the batch variable and tumor stage included as a biological covariate to preserve stage-associated biological variation. All subsequent analyses were performed on batch-corrected, normalized expression matrices. Differential gene expression across clinical stages was assessed using the limma package^69^ with voom transformation and empirical Bayes moderation. Genes with absolute log2 fold change >1 and Benjamini-Hochberg false discovery rate (FDR) < 0.01 in at least one stage comparison were considered significantly differentially expressed. Stage-dynamic transcriptional patterns were further modeled using Mfuzz soft clustering (nine clusters, fuzzifier m = 1.25), and genes with minimum cluster membership >0.7 in clusters showing monotonic trends across stages were selected for downstream analyses. Functional enrichment analyses were performed using clusterProfiler (v4.8.3) for Gene Ontology, KEGG, and Reactome pathways. Immune and stromal cell enrichment was inferred using CIBERSORT, xCell, and ESTIMATE. Pathway activity scores were calculated using GSVA and ssGSEA. Candidate gene selection integrated statistical significance, cluster membership, pathway relevance, and penalized Cox regression using LASSO (glmnet v4.1) with 10-fold cross-validation.

All computational analyses were performed in R (v4.3.1). Data visualization was conducted using ComplexHeatmap and ggplot2. Reproducible analysis scripts and workflows will be deposited in a public repository upon publication to ensure transparency and reproducibility.

#### Expanded analytical framework

To characterize coordinated transcriptional changes across PDAC stages, unsupervised Mfuzz-based soft clustering was used to identify groups of co-expressed genes with dynamic trajectories along disease progression. These clusters were subsequently interpreted through complementary analytical layers, including functional enrichment analyses (GSEA, GO, and KEGG), pathway activity scoring using GSVA and ssGSEA, and computational inference of immune and stromal cell enrichment with xCell, CIBERSORT, and ESTIMATE. Metabolic pathway activity was inferred from transcriptomic data using curated gene sets derived from the RECON2 human metabolic network, enabling comparative assessment of stage-associated metabolic programs without direct measurement of metabolic flux. In parallel, penalized regression approaches (Elastic-Net and LASSO) were applied to derive compact gene panels associated with disease stage and survival. Although these analyses are inherently correlative, their integration enables a systems-level description of tumor-intrinsic signaling, metabolic adaptation, and microenvironmental remodeling across PDAC progression, and provides a framework for generating testable hypotheses for subsequent mechanistic and therapeutic studies.

#### Clustering

Differential expression across disease stages was first assessed using limma, and genes with Benjamini-Hochberg adjusted *p* values <0.01 and absolute log2 fold change >1 in at least one stage comparison were selected for clustering. Stage-dynamic gene expression patterns were modeled using soft c-means clustering implemented in the Mfuzz R package. A fuzzifier parameter (m) of 1.25 was applied, and genes with minimum membership values > 0.7 within clusters displaying monotonic increasing or decreasing trajectories across stages were retained for downstream analyses. Clusters were used to define transcriptional modules for subsequent functional enrichment and pathway analyses.

#### Immune and stromal cell enrichment analysis

Computational inference of immune and stromal cell enrichment was performed on batch-corrected normalized expression data using xCell, CIBERSORT (relative mode, LM22 signature matrix), and ESTIMATE. xCell^61^ was used to obtain enrichment scores for immune and stromal cell populations, CIBERSORT to estimate relative immune cell fractions, and ESTIMATE to compute immune, stromal, and tumor purity scores. For stage-level comparisons, cell-type enrichment scores were summarized by calculating the mean score for each cell type across samples within each disease stage. Statistical comparisons between stages were performed using non-parametric tests with multiple-testing correction, and concordant trends observed across methods were prioritized for biological interpretation.

#### Stage-specific gene signatures and external validation

To derive compact transcriptional signatures representative of early and late disease states, genes were selected from stage-dynamic clusters based on a combination of high Mfuzz cluster membership (>0.7), consistent monotonic expression trends across stages, and biological relevance to epithelial integrity, metabolism, cytoskeletal remodeling, and hypoxia-related pathways. From cluster C1 (progressively downregulated across progression), four genes were selected: CDH12, SLC14A2, FHIT, and IL17RD. From Cluster C2 (progressively upregulated across stages), four genes were selected: RAC3, EPAS1, COQ7, and LOXL1. For each sample, an “early-stage score” and a “late-stage score” were computed as the mean *Z* score of the corresponding gene sets, with *Z* scores calculated within each dataset to ensure comparability across cohorts. Associations between signature scores, disease stage, and overall survival were evaluated using logistic regression for stage discrimination and Cox proportional hazards models for survival. Kaplan-Meier curves were used for visualization by stratifying patients according to median signature scores. External validation was performed in independent RNA-seq datasets from TCGA-PAAD and ICGC PACA-AU, which were processed using the same normalization and scoring procedures as applied to the discovery cohort.

#### PDAC-derived primary cell cultures

Primary PDAC cell cultures were established from patient-derived xenografts (PDXs) as previously described.^70^ Briefly, fresh PDAC tumor samples were implanted subcutaneously into nude mice to generate PDXs. Tumors were enzymatically dissociated using collagenase type V (C9263; Sigma-Aldrich, St. Louis, MO), and isolated cells were cultured in serum-free ductal medium (SFDM) until experimental use. All *in vitro* models used in this study were generated within the PaCaOmics clinical trial (ClinicalTrials.gov identifier: NCT01692873) conducted at the Paoli-Calmettes Institute (Marseille, France). Five cultures were derived from resected primary tumors (PDAC020T, PDAC021T, PDAC025T, PDAC056T, and PDAC079T), five from primary tumors of metastatic PDAC patients (PDAC003T, PDAC014T, PDAC036T, PDAC052T, and PDAC054T), and four from liver metastases (PDAC013T, PDAC031T, PDAC073T, and PDAC089T). Cells were maintained in DMEM/F12 supplemented with 1.22 g/L nicotinamide, 5 g/L glucose, 5% Nu-Serum IV, 0.5% ITS+ Universal Culture Supplement, 1 μM dexamethasone, 10 ng/mL cholera toxin, 50 nM 3,3′,5-triiodo-L-thyronine, 25.2 mg/L bovine pituitary extract, and 20 μg/L epidermal growth factor. Cultures were maintained at 37°C in a humidified incubator with 5% CO_2_.

#### RT-qPCR

Total RNA was extracted using the RNeasy kit (Qiagen), and cDNA synthesis was performed using the GoScript reverse transcription system (Promega) according to the manufacturer’s instructions. Real-time quantitative PCR (RT-qPCR) was performed using the AriaMx system (Agilent). Gene expression was normalized to the housekeeping gene 36B4, and relative expression levels were calculated using the ΔCt method. Primer sequences were as follows: COQ7 (F: TGGAGCAAACCGCATCTATGCC; R: CAAGGGCATCAGAACTGTTGGC); EPAS1 (F: GCGCTAGACTCCGAGAACAT; R: TGGCCACTTACTACCTGACCCTT); RAC3 (F: ACAAGGACACCATTGAGCGGCT; R: CCTCGTCAAACACTGTCTTCAG); FHIT (F: GGACTTTCCTGCCTCTTGGAGA; R: GCGGTCTTCAAACTGGTTGGCA); IL17RD (F: GAATGTCGTCCAGTGTTTCGCC; R: GATGAACTGGGACTCGTGGATC); B2M (F: CCACTGAAAAAGATGAGTATGCCT; R: CCAATCCAAATGCGGCATCTTCA); 36B4 (F: AATCCCTGACGCACCGCCGTGATG; R: TGGGTTGTTTTCCAGGTGCCCTCG).

#### Invasion assay

Transwell migration and invasion assays were performed using 6.4 mm inserts with 8 μm pore-size polyethylene terephthalate membranes (Corning, Cat# 353097) placed in 24-well companion plates (Corning, Cat# 353504). Cells were pretreated with mitomycin C (10 μg/mL) for 2 h to inhibit proliferation prior to seeding. The lower chamber was filled with 500 μL of growth medium containing 10% fetal bovine serum as chemoattractant. A total of 5 x 10^4^ cells per insert were seeded in Matrigel diluted 1:5 in growth medium and allowed to invade for 24 h at 37°C and 5% CO_2_. After incubation, non-invading cells were removed from the upper membrane surface using cotton swabs. Cells on the lower membrane surface were fixed with cold methanol for 15 min at −20°C, stained with 0.4% crystal violet for 30 min, and imaged under a light microscope (10× magnification). Invasive cells were counted in five randomly selected fields per insert. Values represent the mean of five fields per biological replicate.

#### Total ROS and mitochondrial ROS measurement

Cells were seeded at 5 × 10^4^ cells per well in 12-well plates and incubated for 24 h. Cells were then detached, washed with PBS, and incubated with CellROX Green (5 μM; Thermo Fisher, C10444) or MitoSOX Red (5 μM; Thermo Fisher, M36008) diluted in HBSS for 30 min at 37°C in the dark. Excess dye was removed by washing with HBSS, and Hoechst (1:2000) was added to exclude dead cells. Fluorescence was measured using a CytoFLEX S flow cytometer, with at least 10,000 live cells analyzed per sample. Data were analyzed using FlowJo software, and median fluorescence intensity (MFI) values gated on live cells were reported as relative fluorescence units.

#### Oligomycin treatment and viability assessment

Cells were seeded at 5,000 cells per well and allowed to attach overnight. Cultures were treated with vehicle or oligomycin (2 μM) for 24 h under standard culture conditions. Cell viability was estimated by quantifying relative cell number using crystal violet staining, with absorbance measured after dye solubilization. Results were expressed as percentage of viable cells relative to matched vehicle-treated controls.

#### PDAC-derived primary cells cultures

Primary cell cultures derived from PDAC were obtained as previously described.^70^ Briefly, PDAC tumor samples were implanted subcutaneously into nude mice to generate patient-derived xenografts (PDXs). Tumors were then dissociated using collagenase type V (C9263; Sigma-Aldrich, St. Louis, MO), and isolated cells were cultured in SFDM until use. All *in vitro* models used in this study were generated within the PaCaOmics clinical trial (ClinicalTrials.gov identifier: NCT01692873), conducted at the Paoli-Calmettes Institute (Marseille, France). Five samples were obtained from resected tumors (PDAC020T, PDAC021T, PDAC025T, PDAC056T, and PDAC079T), five from primary tumors of metastatic PDAC (PDAC003T, PDAC014T, PDAC036T, PDAC052T, and PDAC054T), and four samples were from liver metastasis (PDAC013T, PDAC031T, PDAC073T, and PDAC089T). Primary human PDAC cell lines were maintained in DMEM/F12 supplemented with 1.22 g/L nicotinamide, 5 g/L glucose, 5% Nu-Serum IV, 0.5% ITS+ Universal Culture Supplement (insulin, human transferrin, and selenous acid), 1 μM dexamethasone, 10 ng/L cholera toxin, 50 nM 3,3′,5-triiodo-L-thyronine, 25.2 mg/L bovine pituitary extract, and 20 μg/L epidermal growth factor. Cells were cultured at 37°C in a humidified incubator with 5% CO_2_.

#### RT-qPCR

Total RNA was extracted from cells using the RNeasy kit (Qiagen), and cDNA was obtained by reverse transcription using the Go Script kit (Promega), according to the manufacturer’s instructions. Real-time quantitative PCR (RT-qPCR) was performed using the AriaMx system (Agilent). Primer sequences are listed below: COQ7: forward: TGGAGCAAACCGCATCTATGCC and reverse: CAAGGGCATCAGAACTGTTGGC; EPAS1: forward: GCGCTAGACTCCGAGAACAT and reverse: TGGCCACTTACTACCTGACCCTT; RAC3: forward: ACAAGGACACCATTGAGCGGCT and reverse: CCTCGTCAAACACTGTCTTCAG; FHIT: forward: GGACTTTCCTGCCTCTTGGAGA and reverse: GCGGTCTTCAAACTGGTTGGCA; IL17RD: forward: GAATGTCGTCCAGTGTTTCGCC and reverse: GATGAACTGGGACTCGTGGATC; B2M: forward: CCACTGAAAAAGATGAGTATGCCT and reverse: CCAATCCAAATGCGGCATCTTCA; 36B4: forward: AATCCCTGACGCACCGCCGTGATG and reverse TGGGTTGTTTTCCAGGTGCCCTCG.

#### Invasion

Transwell migration and invasion assays were performed using 6.4 mm cell culture inserts with 8 μm pore-size polyethylene terephthalate membrane (Corning, Cat# 353097) placed into Falcon 24-well permeable support companion plate (Corning, Cat# 353504). Cells were pretreated with 10 μg/mL of cell cycle inhibitor mitomycin C for 2 h before seeding. The lower chamber was filled with 500 μL of growth medium pH 7.4 containing 10% FBS. The 50 × 10^3^ primary PDAC derived cells/insert were seeded with Matrigel (diluted in growth medium in ratio 1:5. The cells were allowed to migrate or invade through the pores of the insert membrane for 24 h at 37°C and 5% CO_2_. After incubation, cells were washed with PBS, and non-migrating/invading cells were removed from the upper side of the membrane using a cotton swab; cell fixation with cold methanol for 15 min at −20°C was then performed. Cells were stained with 0.4% crystal violet solution at room temperature in the dark for 30 min and imaged under a light microscope (10× magnification). Invasive cells were counted in five representative fields of view. The mean numbers of cells obtained from the five images of each biological replicate were used for statistical analysis.

#### Total ROS and mROS determination

Cells were seeded at 50,000 cells per well in 12-well plates. After 24 h of incubation, the cells were detached with trypsin, collected, and rinsed with PBS. Subsequently, CellROX Green at 5 μM (Thermo Fisher, C10444) or Mitosox Red at 5 μM (Thermo Fisher, M36008) diluted in HBSS were added and incubated for 30 min at 37°C and 5% CO_2_ in the dark. Excess probe was removed by washing the cells with HBSS, and Hoechst (1:2000) was added to stain dead cells. Fluorescence signals were measured using a flow cytometer (Cytoflex S). A minimum of 10,000 cells was analyzed for each primary cells. Data were analyzed using FlowJO software on median fluorescence level gated on live cells and data expressed as relative fluorescence units.

#### Oligomycin treatment and viability assessment

Cells were seeded at 5,000 cells per well and allowed to attach overnight. Cultures were treated with vehicle or oligomycin (2 μM) for 24 h under standard culture conditions. Cell viability was estimated by quantifying relative cell number using 0.4% crystal violet staining, with absorbance measured after dye solubilization. Results were expressed as percentage of viable cells relative to matched vehicle-treated controls.

#### Statistical analysis

Overall survival (OS was defined as the time from diagnosis to death from any cause. Survival curves were estimated using the Kaplan-Meier method and compared using the log rank test. Associations between gene expression, pathway scores, or signature scores and OS were evaluated using Cox proportional hazards regression models. Hazard ratios (HRs) and 95% confidence intervals (CIs) were calculated accordingly. Where indicated, multivariable Cox models were adjusted for tumor stage. For *in vitro* functional assays, comparisons between more than two groups were performed using the Kruskal-Wallis test followed by Dunn’s post hoc multiple-comparison test. When only two groups were compared, the Mann-Whitney U test was applied. P-values were adjusted for multiple testing when appropriate, and statistical significance was defined as adjusted *p* < 0.05 unless otherwise specified.

### Quantification and statistical analysis

Unless otherwise indicated, analyses were performed in **R v4.3.2** with set seed 20251031 for reproducibility. Two-sided tests were used throughout. Multiple testing was controlled by **BH FDR**; adjusted **q < 0.05** was considered significant. Effect sizes (NES, log2FC, HR, ΔAUC) and 95% CIs are reported where applicable. Plots were generated using **ggplot2 v3.5.1** and **ComplexHeatmap v2.18.0**. Asterisks indicate statistical significance levels: *p* < 0.05 (∗), <0.01 (∗∗), and <0.001 (∗∗∗).

### Additional resources

• key resources table lists software, versions, and references.
• Containerized environments (Dockerfiles) and **sessionInfo()** outputs accompany the code repository to ensure exact reproducibility.

#### PDAC-derived primary cell cultures

Primary PDAC cultures were generated from patient-derived xenografts (PDXs) established by subcutaneous implantation of fresh tumor samples into nude mice, as previously described. Tumors were dissociated using collagenase type V (Sigma-Aldrich, C9263), and isolated cells were cultured in SFDM. All models were generated within the PaCaOmics clinical trial (ClinicalTrials.gov: NCT01692873) at the Paoli-Calmettes Institute. Cultures were derived from resected primary tumors (*n* = 5), primary tumors from metastatic patients (*n* = 5), and liver metastases (*n* = 4).

#### Cell culture conditions

Primary PDAC cells were maintained in DMEM/F12 supplemented with 1.22 g/L nicotinamide, 5 g/L glucose, 5% Nu-Serum IV, 0.5% ITS+, 1 μM dexamethasone, 10 ng/mL cholera toxin, 50 nM 3,3′,5-triiodo-L-thyronine, 25.2 mg/L bovine pituitary extract, and 20 μg/L epidermal growth factor, at 37°C in 5% CO_2_.

#### RT-qPCR

Total RNA was extracted using the RNeasy Kit (Qiagen) and reverse-transcribed using GoScript (Promega). Quantitative PCR was performed on an AriaMx system (Agilent). Gene expression was normalized to 36B4 using the ΔCt method. Primer sequences are provided in the original Materials and Methods section.

#### Invasion assays

Transwell invasion assays were performed using 8 μm pore-size inserts (Corning). Cells were pretreated with mitomycin C (10 μg/mL, 2 h), embedded in Matrigel (1:5), and allowed to invade for 24 h toward medium containing 10% FBS. Invading cells were fixed, stained with crystal violet, and quantified in five random fields per insert.

#### Measurement of total and mitochondrial ROS

Cells were stained with CellROX Green (5 μM) or MitoSOX Red (5 μM) in HBSS for 30 min at 37°C in the dark, counterstained with Hoechst, and analyzed using a CytoFLEX S flow cytometer. Median fluorescence intensity of live cells was quantified using FlowJo.

#### Oligomycin treatment and viability assessment

Cells were treated with vehicle or oligomycin (2 μM) for 24 h. Cell viability was estimated by crystal violet staining and expressed relative to matched vehicle-treated controls.

## Supplemental information

Table S1. Differential Gene Expression of Cluster C1

Table S2. Differential Gene Expression of Cluster C2

Table S3. Differential Gene Expression of Cluster C3

Table S4. Differential Gene Expression of Cluster C4

Table S5. Differential Gene Expression of Cluster C5

Table S6. Differential Gene Expression of Cluster C6

Table S7. Differential Gene Expression of Cluster C7

Table S8. Differential Gene Expression of Cluster C8

Table S9. Differential Gene Expression of Cluster C9

Table S10. Differential Gene Expression of Cluster C10
